# Measuring Gambling Reinforcers, Over Consumption and Fallacies: The Psychometric Properties and Predictive Validity of the Jonsson-Abbott Scale

**DOI:** 10.3389/fpsyg.2017.01807

**Published:** 2017-10-16

**Authors:** Jakob Jonsson, Max W. Abbott, Anders Sjöberg, Per Carlbring

**Affiliations:** ^1^Department of Psychology, Stockholm University, Stockholm, Sweden; ^2^Gambling and Addictions Research Centre, Auckland University of Technology, Auckland, New Zealand

**Keywords:** predictive, reinforcers, over consumption, gambling fallacies, CFA, longitudinal, gambling problem

## Abstract

Traditionally, gambling and problem gambling research relies on cross-sectional and retrospective designs. This has compromised identification of temporal relationships and causal inference. To overcome these problems a new questionnaire, the Jonsson-Abbott Scale (JAS), was developed and used in a large, prospective, general population study, The Swedish Longitudinal Gambling Study (Swelogs). The JAS has 11 items and seeks to identify early indicators, examine relationships between indicators and assess their capacity to predict future problem progression. The aims of the study were to examine psychometric properties of the JAS (internal consistency and dimensionality) and predictive validity with respect to increased gambling risk and problem gambling onset. The results are based on repeated interviews with 3818 participants. The response rate from the initial baseline wave was 74%. The original sample consisted of a random, stratified selection from the Swedish population register aged between 16 and 84. The results indicate an acceptable fit of a three-factor solution in a confirmatory factor analysis with ‘Over consumption,’ ‘Gambling fallacies,’ and ‘Reinforcers’ as factors. Reinforcers, Over consumption and Gambling fallacies were significant predictors of gambling risk potential and Gambling fallacies and Over consumption were significant predictors of problem gambling onset (incident cases) at 12 month follow up. When controlled for risk potential measured at baseline, the predictor Over consumption was not significant for gambling risk potential at follow up. For incident cases, Gambling fallacies and Over consumption remained significant when controlled for risk potential. Implications of the results for the development of problem gambling, early detection, prevention, and future research are discussed.

## Introduction

Gambling availability has increased markedly in recent decades (Arvidsson et al., 2016). This increase has been associated with growth in gambling participation and expenditure. In most jurisdictions where general population surveys have been conducted a majority of adults report taking part in one or more gambling activities on an annual or more frequent basis (Williams et al., 2012a). In the most recent Swedish national survey 61% of men and 55% of women participated during the past 12 months (Public Health Agency of Sweden, 2016b). Most people who gamble do so infrequently and/or have low levels of expenditure. A minority has higher levels of engagement and is at greater risk of developing gambling-related problems. The prevalence of gambling disorder or serious problem gambling usually ranges from 0.5 to 3%. Substantially, more people experience some loss of control over gambling and subclinical gambling-related harm (Williams et al., 2012a; Abbott et al., 2014). In Sweden, based on the Problem Gambling Severity Index, 0.4% (95% CI 0.28–0.53%) of adults are estimated to be current problem gamblers, 1.3% moderate-risk gamblers and 4.2% low-risk gamblers (Public Health Agency of Sweden, 2016b). This means that approximately one in 10 gambling participants experience at least some form of reduced control over gambling and/or adverse consequences.

There is no unitary theoretical model for the development of gambling disorder or less serious gambling problems. Clinical and epidemiological studies have found strong associations between involvement in some forms of gambling and problem gambling (Stevens and Young, 2010). Cross-sectional studies have identified additional gambling related factors such as gambling fallacies, gambling behavior, commencing gambling at an early age and experiencing a big win that are associated with problem gambling (Rönnberg et al., 1999; Jonsson et al., 2003; Wardle et al., 2011; Williams et al., 2012a). Some sociodemographic groups including males, young adults, low-income people and single status are almost universally found to be at high risk (Abbott et al., 2013). In Sweden, people born outside the country also have elevated risk (Abbott et al., 2014). In addition to some gambling and sociodemographic factors, there are strong associations with personality characteristics including impulsivity, mental health disorders and substance use and misuse (Bruneau et al., 2016).

The emergence of a body of longitudinal research in the gambling field makes it possible to assess whether or not cross-sectional correlates of problem gambling prevalence precede and predict the development and onset (incidence) of gambling problems. During the past decade five large-scale prospective studies have been conducted (Swedish National Institute of Public Health, 2012, 2013; Billi et al., 2014; Romild et al., 2014; Abbott et al., 2015a,b, 2016; el-Guebaly et al., 2015; Williams et al., 2015). The Swedish and New Zealand studies are still in progress.

The foregoing prospective studies have found that gambling-related factors are the strongest predictors of problem gambling development. These factors include experiencing an early big win, commencing gambling at a young age, having family members who gamble regularly and/or are problem gamblers (in the past and currently), frequency of participation, expenditure, number of forms engaged in and gambling as a favored leisure activity. People who experienced past gambling problems were also prone to relapse. Mental health variables including mental health disorders, substance abuse or dependence and behavioral addictions also predicted future problem gambling. In New Zealand, in addition to gambling-related and mental health factors, ethnicity was a strong risk factor. Maori, Pacific Islanders and Asian people were at particularly high risk. High deprivation, experiencing major life events, lower quality of life and psychological stress were further risk factors and high family income and usually gambling with others were protective (Abbott et al., 2015a,b, 2016). Ethnicity was also a risk factor in the Canadian studies, with non-Caucasians being at higher risk.

The prevention of gambling problems and harm has received increased attention in recent years (see Williams et al., 2012b for an overview). Prevention measures include public awareness raising and education, policy initiatives, restrictions on who can gamble and restrictions and alterations to how gambling is provided. The latter category includes ‘responsible gambling’ measures such as enabling participants to set spending limits and providing feedback on gambling patterns and self-tests for gambling risk or problems.

Early intervention, engaging people before they develop a gambling problem, is an important part of a comprehensive prevention strategy. This calls for the identification of early indicators of problem gambling. As mentioned, heavy gambling engagement is a major risk factor for problem development. To date the role of heavy engagement, consumption and overconsumption in developing problems has not received much attention in its own right (Williams and Volberg, 2014). It has received some consideration as an aspect of loss of control and Currie et al. (2008) have sought to develop low-risk gambling participation limits. The measures include gambling frequency, gambling expenditure and gambling expenditure as a percentage of gross income. While promising, the predictive validity of low-risk limits is yet to be assessed using prospective data.

Gambling-related cognitive distortions and fallacies are relatively commonplace and appear to be risk factors for the development of problem gambling (Leonard and Williams, 2016). Gambling fallacies predicted future problem gambling in the two Canadian prospective studies. Challenging false beliefs about the nature of randomness, over-estimation of skill components in gambling activities and superstitious views about ways to control gambling outcomes through public education campaigns and education programs in schools or at gambling sites may contribute to reducing the incidence of at-risk and problem gambling.

Motives for gambling may also be relevant to problem development and early intervention. As mentioned life events, psychological distress and mental health disorders are risk factors for the development of problem gambling. It is likely that participation in some forms of gambling provide an escape from negative emotions and this could increase gambling exposure and the psychological salience, e.g., negative reinforcement value, of that exposure (Blaszczynski and Nower, 2002). Gambling for escape or distraction was a risk factor for problem development in one of the two Canadian prospective studies. Performance on two Gambling Motives Questionnaire (Stewart and Zack, 2008) subscales, enhancement and emotional coping, have been found to be associated with problem gambling (MacLaren et al., 2014). In a recent Swedish study, moderate risk gamblers participated for challenge and coping reasons more often than low risk gamblers (Sundqvist et al., 2016).

Existing problem gambling screens have covered aspects of gambling fallacies and the reinforcing aspects of gambling, although not used in longitudinal research. The Victorian Gambling Screen includes three items on the enjoyment of gambling among its twenty items (Tolchard and Battersby, 2010). The full Canadian Problem Gambling Inventory has two items on faulty cognitions and three items on gambling as self-medication (Ferris and Wynne, 2001).

One purpose of the Swedish Longitudinal Gambling Study (Swelogs) is to advance understanding of the early development of problem gambling. The research team sought to identify early indicators, examine relationships between indicators and assess their capacity to predict future problem progression. To this end, two team members developed the Jonsson-Abbott Scale (JAS), including items designed to assess the three domains of gambling reinforcements, gambling over-consumption and gambling fallacies (Romild et al., 2014). The theoretical definition of Reinforcers is that the gambling behavior is psychologically reinforcing in some way. The items were chosen to reflect positive reinforcement as excitement and joy, negative reinforcement as forgetting everything else for a while and a socially rewarding aspect. Over consumption is defined as gambling more than intended and experiencing difficulties in refraining from gambling. The items were chosen to mirror that. Gambling fallacies is defined as the misconception that gambling is a way to make money in the long run and that winnings is related to skill. The rationale for developing a new scale was the lack of an existing short screen covering these three areas. Due to restricted space in the interview/questionnaire, there were three to four items chosen for each domain using a consensus process.

In an 11-year follow-up study of lifetime problem gamblers and matched controls (*n* = 423), the three JAS-domains showed significant Pearson r relationships with SOGS-R: gambling reinforcements 0.48, gambling over-consumption 0.52, and gambling fallacies 0.39. Furthermore, the problem gambling group showed significantly higher scores on all three JAS-domains compared with the controls (Public Health Agency of Sweden, 2015).

The aims of this study are to further examine the psychometric properties of the JAS and assess the predictive validity of this new measure. More specifically, it seeks to assess the capacity of identified JAS dimensions to predict increases in problem gambling risk level and problem gambling over 1 year.

## Materials and Methods

### Data Collection

Data were collected within the Swelogs epidemiological track – a prospective study with four waves of data-collection from Swedish citizens aged 16–84 years at baseline. A stratified random sampling procedure was applied for drawing 15000 individuals from the Swedish register of the total population. Data from the two first waves are used in this study. The baseline wave 1, performed between October 2008 and August 2009, included 8165 participants. In wave 2 6021 participants were reassessed between December 2009 and August 2010. The response rate was 55% (8165/15000) in the first wave and 74% (6021/8165) in the second. Interview and questionnaire data were supplemented by register data. Computer-supported telephone interviews were used as the primary method with postal questionnaires used to follow-up those not reached by telephone. Swelogs design, sampling and methodological details are provided in Romild et al. (2014).

### Participants

The 5048 participants (out of 6021) who gambled at least yearly in wave 1 were included in this study. The rationale for this was that only past year gamblers were administered the JAS and PGSI in wave 1. The mean age was 35.2 (*SD* = 19.5) years and 41.7% were women. The sample reduced to 3818 when only participants who reported gambling in both wave 1 and wave 2 were included. The mean age was 36.5 (*SD* = 19.5) years and 40.6% were women.

### Measures

#### Gambling Participation in Wave 1 and Wave 2 – Gambling Risk Potential

Participants were asked about their past 12 months gambling participation in wave 1 and wave 2. Questions covered gambling frequency, time and money spent and modality for nine groups of gambling types. The risk potential for each of the various gambling types was assessed using 7 out of 10 criteria suggested by Meyer et al. (2011). On this basis gambling types were classified as being of low, medium high or high risk (Swedish National Institute of Public Health, 2012). Examples of low risk activities are lotteries (except scratch cards online) and number games at retailers. Medium high risk activities include sports betting (not online), horse betting and online number games. High-risk activities include online bingo, VLTs, casino games and online poker. In the Swelogs study, the medium-high and the high-risk groups both showed distinctly a higher connection with problem gambling than the less than monthly and low-risk-groups that both had very weak connection with gambling problems (Public Health Agency of Sweden, 2016a). Thus, in this study gambling less than monthly was merged with low risk into “Low risk gambling” and medium high and high risk were merged into “High risk gambling.” This reclassification also increased statistical power.

All participants were assigned a risk level based on their highest monthly risk gambling participation in wave 1 and wave 2.

#### Gambling Problem

The Canadian Problem Gambling Severity Index (PGSI) was used in wave 1 and wave 2 to measure gambling problems (Ferris and Wynne, 2001). It employed the response format Never (0), Seldom (1), Often (2), and Always (3). Participants with an overall PGSI score of 0-2 were classified No problem and those with a score of 3-27 were classified Gambling problem.

#### Jonsson-Abbott Scale

The 11 JAS-items were asked in wave 1 only. The directions for objective scale development outlined by Clark and Watson (1995) served as a guide when developing the scale. The items are Likert type with a seven-step response scale ranging from “Do not agree at all” to “Agree completely.” The items (see **Table 1**), are categorized into Reinforcers, Over consumption and Gambling fallacies.

**Table 1:** The Jonsson-Abbott Scale (JAS): items and categories.

Item	Category
(1) I gamble for the excitement	Reinforcer
(2) Gambling is among the most enjoyable things there are	Reinforcer
(3) Gambling can make me forget everything else for a while	Reinforcer
(4) My gambling gives me friends	Reinforcer
(5) I gamble for more money than intended	Over consumption
(6) I gamble a longer time than intended	Over consumption
(7) I gamble when I should have done other things	Over consumption
(8) When gambling, I find it hard to stop	Over consumption
(9) My gambling is a way to make money	Gambling fallacy
(10) When I win, it is due to my skill	Gambling fallacy
(11) If I just gamble enough, my gambling will pay off	Gambling fallacy

### Analysis

We investigated if the scales represented three different constructs by subjecting the items to a confirmatory factor analysis (CFA; Bollen, 1989). The postulated three-factor representation of the 11-item gambling scale was empirically tested using the CFA procedures with maximum likelihood estimation in Lavaan (Rosseel, 2012). To evaluate model fit the likelihood-ratio χ^2^ test, the Root-Mean-Square Error of Approximation (RMSEA), the Comparative Fit Index (CFI), the Normed Fit Index (NFI) and the Tucker-Lewis Index (TLI), were used. Browne and Cudeck (1993) suggested that RMSEA values of 0.08 or less indicate reasonable error of approximation in relation to the degrees of freedom, while values of 0.05 or less indicate close fit. We relied on MacCallum et al. (1996) suggestion to use 0.01, 0.05, and 0.08 to indicate excellent, good, and mediocre fit, respectively. The recommended cut-off values of TLI, CFI, and NFI are 0.95 or higher (Hu and Bentler, 1999).

To address the question concerning predictive power of JAS we used logistic regression analyses (Menard, 2002) with gambling risk potential and incident cases as dependent variables and the three factors (i.e., Reinforcer, Over consumption, Gambling Fallacy) as predictor variables. The likelihood ratio test was used to test our models. It is a test of the significance of the difference between the likelihood ratio (-2 log likelihood) for our model with predictors (called model chi square) minus the likelihood ratio for baseline model with only a constant in the model. Significance at the 0.05 level or lower means that the model with the predictors is significantly different from the one with the constant only (all ‘b’ coefficients being zero). It measures the improvement in fit that the explanatory variables make compared to the null model. Chi square is used to assess significance of this ratio. Akaike’s Information Criterion (AIC) and the Bayesian Information Criterion (BIC) were also used to evaluate the models. Both are based on -*2 Log Likelihood*. The value of AIC and BIC can be used to compare various models for the same data set to determine the best-fitting model. The model having the smallest value is usually preferred (Akaike, 1974; Kass and Wasserman, 1995). Both unstandardized (B) and standardized coefficients (β) are reported in the logistic regression analyses (Menard, 2011).

### Ethics Statement

The original Swelogs study plan was approved by the Regional Ethical Review Board in Umeå in 2008 (Dnr 08-78). Additional ethical applications have been submitted in subsequent years due to changes in questionnaires for consecutive data collections. For this study an ethical application for secondary analysis was submitted (Dnr 2016/410-32). All submitted applications have been approved.

## Results

In **Table 2** the CFA results (i.e., standardized factor loadings) for the proposed three factor model are presented. The results of the CFA analysis indicate a mediocre fit between the three-factor model and the data (χ^2^[41] = 1077.742; *p* < 0.001; RMSEA = 0.071; *p* < 0.05; 90% CI [0.067, 0.075]; CFI = 0.939; TLI = 0.918; NFI = 0.94). Closer examination of the modification indices of the CFA showed that item 10 “When I win, it is due to my skill” loaded on two latent factors. When that path was freed, the modified model indicated a significantly better fit (χ^2^[40] = 665.356; *p* < 0.001; χ^2^ diff[1] = 412.390; *p* < 0.001; RMSEA = 0.056; *p* < 0.05; 90% CI [0.052, 0.060]; CFI = 0.963; TLI = 0.949; NFI = 0.936). The results suggest that this item should be included in both factors, removed from the scales, or reformulated. Overall, the results confirm that these three constructs are empirically separated because of the specific variance of each factor, in other words, the corrected for attenuation correlations are far from 1.00. We also contrasted our two proposed three factor models with a one factor solution (χ^2^[44] = 1077.742; *p* < 0.001; RMSEA = 0.105; *p* < 0.05; 90% CI [0.101, 0.108]; CFI = 0.857; TLI = 0.821; NFI = 0.854) which showed a worse fit when both our original model was compared (χ^2^ diff[3] = 1393.671; *p* < 0.001) and when our modified model was compared (χ^2^ diff[4] = 1816.052; *p* < 0.001).

**Table 2:** Standardized factor loadings based on confirmatory factor analysis.

	F1	F2	F3
(2) Gambling is among the most enjoyable things there are	0.71		
(3) Gambling can make me forget everything else for a while	0.70		
(1) I gamble for the excitement	0.54		
(4) My gambling gives me friends	0.42		
(6) I gamble a longer time than intended		0.82	
(5) I gamble for more money than intended		0.74	
(8) When gambling, I find it hard to stop		0.68	
(7) I gamble when I should have done other things		0.66	
(10) When I win, it is due to my skill			0.52
(9) My gambling is a way to make money			0.63
(11) If I just gamble enough, my gambling will pay off			0.70

*F1 = Reinforcer; F2 = Overconsumptions; F3 = Gambling Fallacy. The latent factor correlation between F1 and F2 was 0.73, between F1 and F3 0.69, and between F2 and F3 0.69*.

The internal consistency reliability (Chronbachs’s alpha) of the three scales Reinforcers, Over consumption and Gambling fallacies are 0.67, 0.82, and 0.58, respectively. The overall internal consistency of the JAS scale is 0.83. The correlation between JAS (all items) and PGSI is *r* = 0.49 and the correlations between subscales and PGSI are Reinforcers 0.34, Over consumption 0.59, and Gambling fallacies 0.36.

To investigate the predictive validity of the JAS scale two logistic regression analyses were conducted. The statistical significance of individual regression coefficients (i.e., βs) was tested using the Wald chi-square statistic. From **Table 3** it is evident that in step 1 Gambling fallacies, Reinforcers and Over consumption were significant predictors of risk potential (*p* < 0.05). Gambling Fallacies and Over consumption were also significant predictors of incident cases (*p* < 0.05) and Reinforcers showed a tendency of significance (*p* = 0.053). In the second step, risk potential measured at time 1 was added to the equation. In this second step, the predictor Over consumption was not significant with respect to risk potential. Further, Reinforcers was not a significant predictor of incident cases at time 2. The results suggest that the model with three predictors should be applied to the data (*p* < 0.05). Both AIC and BIC showed better values for the proposed model with the three predictors than for the models using only the constants as predictors.

**Table 3:** Logistic regression analyses (*n* = 3818).

	Risk potential time 2		Incident cases time 2	
	Step 1	Step 2	Step 1	Step 2
**Predictors**	**B (β)**			
Gambling fallacy	0.26^∗∗∗^ (0.06)	0.16^∗∗∗^ (0.07)	0.25^∗∗^ (0.12)	0.22^∗^ (0.04)
Reinforcer	0.37^∗∗∗^ (0.09)	0.19^∗∗∗^ (0.08)	0.20 (0.14)	0.15 (0.03)
Over consumption	0.15^∗∗^ (0.03)	0.05 (0.02)	0.26^∗^ (0.08)	0.25^∗^ (0.04)
Risk potential time 1	–	2.327^∗∗∗^ (0.35)	–	0.61^∗∗^ (0.04)
-2 Log likelihood	3913.318	3220.950	920.197	912.962
AIC	3921.318	3230.950	928.197	922.962
BIC	3946.304	3262.184	954.052	922.962
Nagelkerke	0.12^∗∗∗^	0.35^∗∗∗^	0.05^∗∗∗^	0.06^∗∗∗^

^∗^*p < 0.05, ^∗∗^*p* < 0.01, ^∗∗∗^*p* < 0.001. Standardized coefficients (beta weights) within parentheses were computed with formula 5 reported in Menard (2004). AIC, Akaike information criterion; BIC, Bayesian information criterion*.

## Discussion

The current study examined the dimensionality and predictive validity of JAS. A three-factor model was confirmed by CFA and the subscales of JAS were found to have moderate to high internal consistency. Reinforcers, Over consumption and Gambling fallacies were significant predictors of gambling risk potential and Gambling fallacies and Over consumption were significant predictors of problem gambling onset (incident cases) at follow-up. When controlling for gambling risk potential at baseline, the dimension Overconsumption was no longer significant in predicting risk potential. This is not unexpected given that high gambling risk potential at baseline is a strong predictor of a high gambling risk potential at follow-up, and regular participation in high risk gambling activities has a connection with overconsumption as an early sign of problem gambling. For incident cases, Gambling fallacies and Over consumption were still significant when controlling for risk potential. This is consistent with findings from recent longitudinal gambling research. While various aspects of gambling behavior are strong predictors of problem gambling development, other factors including gambling fallacies, psychological distress and disorders, addictions, personality attributes and sociodemographic factors also have an influence (Swedish National Institute of Public Health, 2012, 2013; Billi et al., 2014; Williams et al., 2015; Abbott et al., 2016).

In this study, high-risk gambling potential level and problem gambling are partly explained by different variables. These results appear to fit well with the etiological model suggested by Williams et al. (2015). They also highlight the role of reinforcements in starting to gamble at a higher risk-level – something not covered in the etiological model. This matter requires further investigation.

Given that the three JAS subscales and regular participation in high risk gambling activities predicted the onset of future moderate risk and problem gambling, these measures are likely to be important in the detection of early problem gambling development. They reflect behaviors and beliefs that could provide a focus for problem gambling prevention programs. Programs could include education to counter gambling fallacies and a variety of policy, regulatory and other measures to prevent and reduce overconsumption.

There is a lack of brief multidimensional screens covering factors relevant to the development of gambling problems. JAS contributes to extant research in the field by providing, in a relatively brief format, a measure with three dimensions that are theoretical and empirical risk-factors for and early signs of problem gambling. This makes JAS suitable for use in longitudinal research. It may also inspire the development of new scales that assess these dimensions more fully.

### Strengths

One strength of this study is that it draws on data from on a large, random general population sample that is nationally representative. Additionally, it is prospective, had relatively low attrition and involved repeated assessment of the same participants 12 months apart. This is a prerequisite for assessing a scale’s predictive validity. The response format used in JAS gave the possibility to respond to the statements in a more nuanced way. This is an asset for the CFA in that it increases response variation.

### Limitations

The study has a number of limitations related to the design and choices made due to limited statistical power. One is the use of PGSI ≥ 3 as an indicator of problematic gambling. The conventional cut score for problem gambling is ≥ 8, although Williams and Volberg (2014) have made a strong case for using ≥ 5 instead. Collapsing gambling participation risk categories is another weakness. Measurement invariance between subgroups in age and gender was not controlled for and attenuation, due to relatively low reliability in two of the JAS dimensions, will have underestimated their predictive capacity. Additionally, the JAS was administered at baseline only. Consequently, it was not possible to look at the dimensions’ stability and how they varied along with change in gambling behavior. Another limitation is that the reliability of JAS is not yet fully explored (e.g., test–retest).

The correlations between two JAS subscales and PGSI were moderately low. However, the PGSI is a unidimensional measure (Miller et al., 2013) whereas the JAS was designed to assess three distinct factors considered likely to be involved in the early development of problem gambling. The JAS, in contrast to the PGSI, was not intended to measure problem gambling *per se*. While overlap between JAS and PGSI performance, administered at the same time, was anticipated, it was expected to be low to moderate.

### Future Research

Problem gambling prevention requires more research, perhaps especially with regard to early interventions. Based on the study findings further investigation is called for on the role of Over consumption, Reinforcers and Gambling fallacies in progression to higher risk gambling levels and the development of gambling problems. This could include examining relationships between these and other relevant constructs and how these relationships change over time with at-risk and problem gambling. Ideally such studies would extend well beyond 12 months.

The JAS could be enhanced by the addition of supplementary items and assessing its psychometric properties in a variety of settings. The reliability of JAS also needs to be further examined. Offering the JAS or similar instruments at gambling sites combined with interventions such as feedback on consumption and/or the facility to pre-commit to gambling expenditure limits warrant investigation.

## Availability

The JAS is freely available for use in research and clinical practice. At time of writing, the instrument has been translated by professional translators into English and is also available in Swedish. Current versions are available at http://spelforskning.se/jas.

## Author Contributions

JJ and MA participated in data collection. JJ and AS did the data analysis. JJ, MA, and AS drafted the manuscript. All authors have read and suggested improvements in several iterations of the manuscript preparation.

## Conflict of Interest Statement

JJ and MA are members of the Swelogs advisory board. JJ is an employee at Sustainable Interaction, a private company working with responsible gambling consultancy and online training with the gambling industry. JJ is involved in ongoing research at the Norwegian gambling operator Norsk Tipping. PC has been the primary investigator of two larger treatment studies on pathological gambling funded by the Public Health Agency of Sweden. He has also received 3-year funding from FORTE, a government agency under the Swedish Ministry of Health and Social Affairs, for an Internet-delivered treatment for concerned significant others of people with problem gambling. In addition, he has received two research grants (Svenska spel and PAF) specifically devoted to only cover the university costs of employing two Ph.D.-students. Both Ph.D.-positions were publically announced and applicants were independently assessed by three reviewers. Finally, PC and JJ has served as an unpaid gambling expert for the National Board of Health and Welfare (Socialstyrelsen) which is a government agency in Sweden under the Ministry of Health and Social Affairs. The other author declares that the research was conducted in the absence of any commercial or financial relationships that could be construed as a potential conflict of interest.
